# IAPs on the move: role of inhibitors of apoptosis proteins in cell migration

**DOI:** 10.1038/cddis.2013.311

**Published:** 2013-09-05

**Authors:** T K Oberoi-Khanuja, A Murali, K Rajalingam

**Affiliations:** 1 Emmy Noether Group of the DFG, Institute of Biochemistry II, Goethe University Medical School, Frankfurt, Germany

**Keywords:** IAPs, XIAP, cIAP1 BIR, tumour, migration, metastases

## Abstract

Inhibitors of Apoptosis Proteins (IAPs) are a class of highly conserved proteins predominantly known for the regulation of caspases and immune signaling. However, recent evidence suggests a crucial role for these molecules in the regulation of tumor cell shape and migration by controlling MAPK, NF-*κ*B and Rho GTPases. IAPs directly control Rho GTPases, thus regulating cell shape and migration. For instance, XIAP and cIAP1 function as the direct E3 ubiquitin ligases of Rac1 and target it for proteasomal degradation. IAPs are differentially expressed in tumor cells and have been targeted by several cancer therapeutic drugs that are currently in clinical trials. Here, we summarize the current knowledge on the role of IAPs in the regulation of cell migration and discuss the possible implications of these observations in regulating tumor cell metastases.

## Facts

- IAPs directly regulate cell shape, migration and tumor metastases.
- IAPs have pro- and anti-migratory roles depending on the cellular context.
- IAPs can regulate the plasticity of tumor cell migration by affecting the stability and activation of Rho GTPases.
- IAPs have been correlated with both negative and positive tumor prognosis.

## Open Questions

- What is the role of IAPs in regulating physiological forms of cell migration like wound healing?
- What are the distinct roles of different IAPs in tumor metastasis?
- What are the various signaling pathways activated by IAP antagonists in the context of tumor cell invasion?

## Inhibitor of Apoptosis Proteins (IAPs)

Inhibitor of apoptosis proteins (IAPs), also known as BIRCs (BIR domain containing proteins) are a class of proteins characterized by the presence of Baculovirus IAP Repeat (BIR) domain, a Zn^2^^+^ ion coordinating protein–protein interaction motif. They are highly conserved from viruses to mammals and were discovered in a genetic screen of baculoviral genome as inhibitors of host programmed cell death machinery, hence named IAPs.^1, 2^ However, recent advances in the field of IAP/BIRC biology strongly establish these proteins to be pivotal for numerous cellular signaling networks. Studies from other organisms down the evolutionary ladder have also revealed that IAPs are pleotropic proteins and not just inhibitors of caspases. Certain prokaryotes, yeasts and plants undergo a form of programmed cell death (PCD) by caspase homologs known as metacaspases.^3^ Yet no IAP homologs are found in prokaryotes and plants; and metacaspases are also not essential for prokaryotic PCD. In case of *S. cerevisiae*, the only known IAP, BIR1p inhibits apoptosis in a YCA1 (*S. cerevisiae* metacaspase)-independent manner. BIR1p is required for proper chromosome segregation and cytokinesis and moreover, YCA1 also has roles in cell cycle regulation, clearance of protein aggregates, and so on.^4, 5, 6^ Further, though there are viral IAP homologs, caspase homologs have not been identified in viruses.^1, 2^

**Figure 1:**
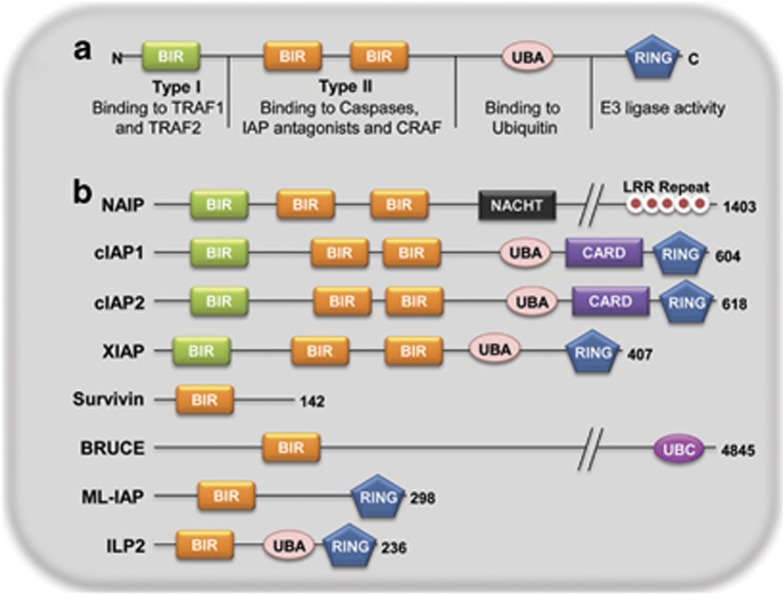
Domain architecture of mammalian IAPs. (**a**) A prototypical IAP is depicted consisting of Type I and Type II BIR domains, ubiquitin-binding domain and RING domain with their functions and molecular structures. (**b**) Various mammalian IAPs are depicted with their known functional domains. BIR, baculoviral IAP repeat domain; NACHT/NOD, nucleotide binding and oligomerization domain; LRR, leucine-rich repeats; UBA, ubiquitin-associated domain; CARD, caspase recruitment domain; RING, really interesting new gene domain; UBC, ubiquitin conjugating domain

There are eight known mammalian IAPs/BIRCs (Figure 1): BIRC1 (neuronal IAP/NAIP), BIRC2 (cellular IAP1/cIAP1/HIAP2), BIRC3 (cellular IAP2/cIAP2/HIAP1), BIRC4 (X-linked IAP/XIAP/hILP), BIRC5 (Survivin), BIRC6 (BIR containing ubiquitin conjugating enzyme/BRUCE/Apollon), BIRC7 (Melanoma IAP/ML-IAP/Livin) and BIRC8 (IAP-like protein 2/hILP2/Ts-IAP). Apart from the BIR domains, there are various other structural motifs defining the characteristics of IAPs. NAIP has a NACHT domain and leucine-rich repeats (LRR) implicated in Nod-like receptor (NLR)-mediated innate immunity signaling.^7^ Another important domain is the really interesting new gene (RING) domain present in cIAP1, cIAP2, XIAP, ML-IAP and hILP2. This domain provides an E3 ubiquitin ligase activity to the proteins by virtue of which, it can promote attachment of monomeric ubiquitin or ubiquitin chains of various kinds to the substrate. RING domains of IAPs have been shown to be responsible for auto-ubiquitination, cross-ubiquitination of other IAPs and substrate ubiquitination. cIAP1, cIAP2, XIAP and hILP2 also have an ubiquitin-associated domain (UBA).^8, 9^ UBA domain binds to monomeric ubiquitin as well as ubiquitin chains helping in protein complex assembly to promote various cell signaling events.^10^ BRUCE lacks both the RING as well as UBA domains but has an ubiquitin conjugation or UBC domain catalyzing the conjugation of ubiquitin to the substrate. cIAP1 and cIAP2 have a CARD domain (CAspase Recruitment Domain), which is also present in various components of apoptotic and inflammation signaling complexes like caspases (-1/-2/-4/-5/-9 and -13), Apaf-1, PIDD, RAIDD, NODs and RIP2. The role of CARD domain in IAPs has been unknown, though a recent study suggested its role in autoinhibition of the RING domain activity of cIAP1^11^ (Figure 1).

As stated above, IAPs can be regulated by auto-ubiquitination and cross-ubiquitination via their RING domains. It has been reported that loss of one IAP leads to an increase in the protein levels of another IAP.^12^ Further, the RING domain of cIAP1 has been reported to mediate the degradation of other RING-bearing IAPs.^13^ In these lines, IAPs have been shown to exist as heteromeric complexes in the cytosol of many human cell lines.^14^ Further, a heteromeric complex of surivivin and XIAP has been shown to resist apoptosis.^15^ IAPs can also be regulated by certain endogenous inhibitors of IAPs like Smac/DIABLO, Omi/HtrA2, XAF1, ARTS and TWEAK.^16, 17, 18, 19, 20^ Smac/DIABLO, the most well-studied IAP inhibitor is a mitochondrial protein released into the cytosol during apoptotic induction. Upon release into the cytosol, Smac/DIABLO binds directly to XIAP by its N-terminal, IAP-binding motif (IBM). IBM interacts with BIR2 and BIR3 of XIAP, and thereby disrupts the function of XIAP to block caspase-3, -7, and -9.^21^ Smac has also been shown to trigger auto-ubiquitination of cIAPs, XIAP and ML-IAP leading to proteasomal degradation of cIAPs but not XIAP and ML-IAP.^22^

**Figure 2:**
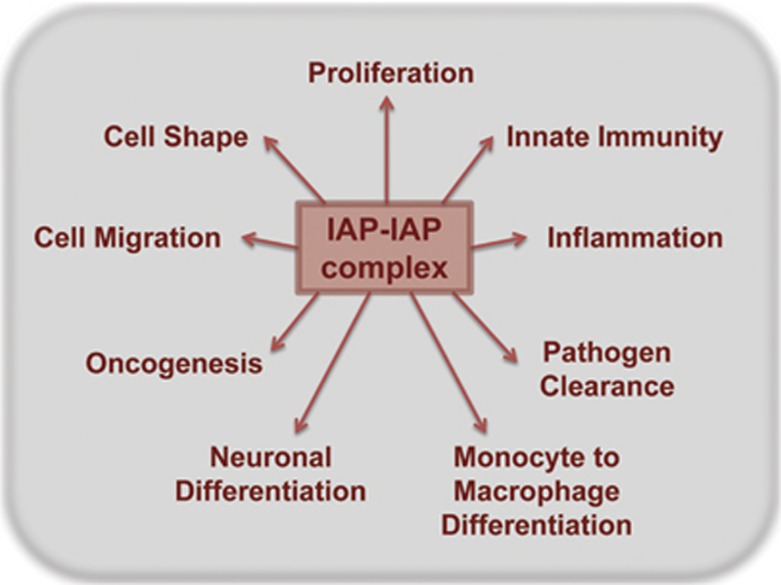
Cellular phenotypes mediated by IAP–IAP complexes. Depicted are the known cellular phenotypes that are regulated by IAPs. IAPs can form homomeric or hetermeric complexes within the cells and have a central role in various cellular signaling pathways that can be translated to various phenotypes

IAPs have come forth as vital signaling molecules at the crux of various cell death and survival pathways. Apart from apoptotic-caspase cascade, IAPs are essential modulators of innate immunity signaling, canonical and non-canonical NF-*κ*B pathways as well as TGF*β* signaling pathway^23^ (Figure 2). Their role as tumor modulators has been substantiated by differential expression of IAP family members in various malignancies. Elevated expression of IAPs in certain tumor types has been correlated with tumor survival and resistance to chemotherapy. So, a variety of antitumor therapeutics, especially small-molecule inhibitors against IAPs (IAP antagonist compounds (IAC), Smac mimetics) are being designed and clinically tested.^24, 25^ Though several studies have demonstrated elevated levels of multiple IAPs in an array of human cancers as well as tumor models, a clinical correlation could not be established for many of them. Furthermore, the relative contribution of individual IAPs towards tumorigenesis and progression is far from clear. There have been opposing prognostic implications for IAPs in different tumor types, strongly suggesting that the role of IAPs in tumorigenesis is context- and cell type-dependent.

## Role of IAPs in Tumorigenesis and Tumor Progression

### IAPs correlated with negative prognosis

IAPs are overexpressed in a number of tumors and are recognized to regulate carcinogenesis at various stages. cIAP1 and cIAP2 have been suggested as proto-oncogenes by various genetic studies. cIAP1/2 and YAP (yes-associated protein) are a part of 11q21–11q23 amplifications observed in a variety of cancers like hepatocellular carcinoma, medulloblastoma, glioblastoma, gastric carcinoma, non-small cell lung carcinoma and osteosarcoma.^26, 27, 28, 29^ Deletions of CARD and RING domains in cIAP2 are linked to generation of an oncoprotein in MALT (mucosa-associated lymphoid tissue) lymphoma.^30^ cIAP2, along with BAX and BMF has been proposed as a three-gene signature for acute myeloid leukemia (ALL) prognosis but there have been contrasting reports for the importance of elevated levels of IAPs in other forms of leukemias.^31, 32, 33, 34^ Elevated levels of IAPs in bladder cancer and colorectal carcinoma correlated with progressive stages of tumor as well as decreased overall patient survival.^35, 36, 37, 38^ In breast carcinoma, higher nuclear expression of XIAP correlated with lower patient survival.^39^ Similarly, in cervical squamous cell carcinoma, recurrence-free survival rates were lower with high levels of nuclear cIAP1.^40^ Furthermore, enhanced expression of survivin, cIAP1, cIAP2 and XIAP has been observed in chemotherapy-induced multi-drug resistant multiple myeloma.^41^ Apart from IAPs, pro-apoptotic, endogenous IAP antagonists like Smac, Omi/HtrA2, XAF1 and ARTS have also been characterized for cancer prognosis.^42, 43, 44, 45^ Independent studies have shown that patients expressing higher levels of Smac have higher chances of recurrence-free survival in AML, breast cancer, NSCLC, RCC, bladder carcinoma and colorectal carcinoma, whereas lower levels have been associated with tumor metastasis and lower life expectancy.^46, 47, 48, 49, 50^ Studies with XAF1 confirmed its prognostic value in pancreatic cancer, squamous cell lung cancer and ovarian cancer.^51, 52^ Low expression levels of XAF1 could be correlated with disease stage, differentiation status and lymph node metastasis in squamous cell lung cancer patients.^53^ Further, studies carried out by Elhasid *et al.* found a loss of ARTS protein expression in all malignant lymphoblasts in a majority of pediatric ALL patients, which was found to be reversed upon remission in almost all of them^54^ (Table 1).

### IAPs correlated with positive prognosis

There have also been reports showing favorable cancer prognosis with elevated levels of IAPs. In case of NSCLC, a study by Ferreira *et al.*^55^ reported that high XIAP levels correlated with lower proliferation rate and longer patient survival, suggesting it as a positive prognostic factor for NSCLC. Elevated ML-IAP expression has been determined as an independent favorable prognosis factor for pediatric ALL as well as relapse-free survival in RCC.^56, 57^ In these lines, a recent study by Lazar *et al.*^58^ showed that high ML-IAP levels correlated with poor prognosis in patients that responded to an autologous melanoma vaccine. On the contrary, in patients that remained with active disease after treatment with melanoma vaccine, low to intermediate levels rather than absence of protein is associated with favorable prognosis due to paradoxical pro-apoptotic ML-IAP activity.^20, 58^ Furthermore, higher levels of IAPs (BIRC1-5) were reported in prostate cancer cell lines as well as prostate cancer samples without any clinical correlation.^59, 60^ However, some recent studies have identified XIAP expression as an independent favorable prognostic marker for longer relapse-free survival in prostate cancer patients (Table 2). IAPs have been recently recognized as crucial regulators of tumor cell migration and metastases.^61^ We will be mainly focusing further on IAP-mediated cellular migration in physiological as well as patho-physiological context in this review.

## Cell Migration

Migration is a complex and cell–type dependent fundamental process that underlies key biological phenomena like tissue formation and maintenance, regeneration as well as pathological conditions like cancer metastasis. While there are various factors that influence cell shape and migration like cell–cell and cell–matrix adhesions, pericellular proteolysis, polarity, as well as the extracellular matrix (ECM), the basic cellular feature that drives this process is the actin cytoskeleton. Seminal work by Lauffenburger and Horwitz^62^ in 1996 proposed a five-step process for cell migration. The process of cell migration begins with protrusions formed by cell at the leading edge in response to an appropriate migration stimulus. This leads to the formation of focal adhesion (FA) complexes that help attach the cell to the ECM. In the event of secretion of proteases, matrix degradation occurs at the leading edge of the cell. This is followed by actomyosin contractility and finally the detachment of the rear end of the cell.^62^ While most of the studies on cell migration were performed in 2D matrices and substrates, recent studies with 3D matrices revealed that cells exploit a much greater range of protrusive structures to migrate through these matrices like filopodia and lobopodia.^63^ This accounts for some of the conflicting results obtained depending on the model system selected to study cell migration. Therefore, care should be taken to interpret the results from dissimilar model systems.

Owing to the integral role of the actin cytoskeleton in migration of cells, Rho GTPases, which are known to regulate many aspects of intracellular actin dynamics, have been highly implicated in this process. Rho GTPases are a family of 20 proteins, among which Rac1, RhoA and Cdc42 are the best studied. Seminal studies showed that each of these proteins have a role in actin cytoskeleton rearrangement; Rac1 was linked to the formation of lamellipodia, Cdc42 to filopodia and RhoA to stress fiber formation by generation of actomyosin contractility.^64^

Cells can migrate as single cells or as a group in clusters, sheets; this has led to the classification of cell migration into single-cell and collective cell migration. Single-cell migration can further be divided into mesenchymal and amoeboid forms of migration. Mesenchymal migration is characterized by an elongated morphology, high attachment of cells to the ECM via FA complexes, as well as secretion of proteases to degrade the ECM. Amoeboid movement is largely independent of cell–matrix contact and proteolytic degradation and the cells show a rounded morphology. Rac1 and Cdc42 have an important role at the leading edge of the cell in mesenchymal form of migration. Rac1 helps to initiate lamellipodium formation and Cdc42 maintains the directionality of the cell.^65, 66^ RhoA is usually detected at the trailing edge of these cells where it facilitates in tail retraction. RhoA has a more prominent role in amoeboid movement via actomyosin contractility through myosin activation.^67, 68^ Cdc42 is also implicated in amoeboid migration and invasion in melanoma cells.^69^

While cells preferentially employ one of the above-described migration types, they have the ability to adapt to the changes in their microenvironment and switch from one mode of migration to the other. Thus, plasticity of cell migration has ample physiological and patho-physiological relevance. Rho GTPases have an integral role in this switch, with their activity often corresponding to the preferred mode of migration of a particular cell type. For example, RhoA activation is associated with amoeboid form of migration and Rac1 with mesenchymal mode of migration, although RhoA-mediated actomyosin contractility is also an essential component of mesenchymal migration contributing to tail retraction. Recent studies have also implicated other Rho GTPases in these processes; RhoE, for example, is downregulated in hepatocellular carcinoma and this is associated with poor prognosis due to loss of E-Cadherin at junctions, thus leading to tumor progression and metastasis.^70, 71^

## Role of IAP-Mediated Signaling in Cell Migration

### IAPs in physiological forms of migration

The first evidence for the role of IAPs in cell migration was demonstrated by Geisbrecht and Montell in 2004 during their studies with border cell migration in *Drosophila* ovaries, an important phenomenon for oogenesis during embryonic development. The process of border cell migration exemplifies how cells of epithelial origin acquire migratory properties. They reported that expression of dominant-negative Rac1 (Rac1-N17) in border cells inhibits border cell migration, and hence, renders the fly infertile. Further, in a forward genetics screen, they found that when overexpressed, DIAP1 or *Drosophila* IAP1 can suppress Rac-N17-mediated border cell migration defect. This effect was mediated by the BIR domains of DIAP1 and was independent of its anti-apoptotic activity as well as its RING domain.^72^ Furthermore, DIAP1 was found to interact with profilin, an actin-binding protein and binding partner for Rac1. This suggests a pro-migratory role of DIAP1 in actin polymerization via profilin to mediate border cell migration in *Drosophila* ovaries. In these lines, we have uncovered that XIAP and cIAP1 can function as the direct E3 ubiquitin ligase of Rac1.^73^ Loss of these two IAPs stabilizes Rac1 and promotes an elongated-mesenchymal mode of migration in many primary and tumor cell lines (discussed below). This observation is in contradiction with the results found with DIAP1, but whether mammalian IAPs can bind to profilin and the physiological relevance of this interaction still need to be tested.

In collaboration with Reinhard Köster's group, we have shown a role for DrXIAP in migration of cerebellar granule neurons (CGNs) during cerebellar development in *Danio rerio*. We employed transgenic zebrafish strain to generate conditional expression of transgenes in the cells of the upper rhombic lip. Enhanced expression of DrXIAP in CGNs lead to a loss of cell polarity and cells were often found in the fourth ventricle forming clusters. These effects were Rac1-dependent as increasing the expression of wild-type Rac1 with DrXIAP could rescue the phenotype.^73^ Thus, DrXIAP shows an anti-migratory effect on progenitor cell migration during zebrafish brain development by regulating Rac1. We also observed that XIAP knockdown in immortalized cerebellar granular cells derived from mice showed enhanced Rac1 levels, suggesting an evolutionarily conserved role for IAPs in controlling Rac1 homeostasis.

IAPs have also been shown to modulate endothelial cell migration. Kim *et al.*^74, 75^ found a role of XIAP in regulating endothelial cell survival and migration. They found that caveolin1 can bind to XIAP via its BIR3 domain, and thus, recruits XIAP to *α*5–integrin complex. XIAP also recruits FAK to this complex and was found to be essential for shear-stress-induced, Src-mediated Y567 phosphorylation of FAK and ERK1/2 activation.^76^ They also showed that XIAP could directly bind to FAK via FRNK domain of FAK and promote endothelial cell migration by inhibiting FRNK-mediated cell adhesion.^77^ As Rac1, FAK and integrins are vital to the dynamics of all forms of cell adhesion and migration, it is tempting to investigate the role of IAPs in other physiological forms of cell migration including wound healing and in immune cell responses. IAPs were shown to exhibit both pro- and anti-migratory functions in tumor cells, which have been discussed below.

### Pro-migratory effects of IAPs in tumor cells

A recent study by Lopez *et al.*^11^ suggested that CARD domain of cIAP1 prevents its auto-ubiquitination through intramolecular inhibition of RING dimerization and hence its E3 ubiquitin ligase activity. They further showed that CARD-mediated RING regulation had implications on cell proliferation and migration. cIAP1 cooperates with Myc to drive cell proliferation by ubiquitination and degradation of myc antagonist, Mad1. By means of reconstitution studies in cIAP1/2 DKO MEFs they could observe more RING activity in CARD deletion mutant of cIAP1 as compared with WT cIAP1. Using the same strategy with scratch assays, they observed enhanced migration in mutant cIAP1 reconstituted cells as compared with WT cIAP1 cells. This suggests a role for CARD domain in regulating cIAP1-mediated cell migration. Furthermore, they demonstrated a loss of wound-healing ability in MCF-7 cells upon cIAP1 knockdown, suggesting a pro-migratory role of cIAP1 in these cells. Though this study finds a role for CARD domains in regulation of RING activity and migration, there have been other studies using IAC-mediated degradation of cIAP1 that suggest a different mechanism of regulation of cIAP1 stability. Report from Blankenship *et al.*^8^ using cIAP1 WT and its CARD deletion mutant suggest that the linker region between BIR3 and CARD domain of cIAP1 and not the CARD domain *per se* is important for modulating its stability. Studies by Fairbrother *et al.*^24^ suggest the role of BIR3–RING interactions in the modulation of RING E3 ligase activity of cIAP1.^78^

Another study by Liu *et al.*^79^ attempts to define the role of XIAP–RING domain in cancer cell migration. It was shown that depletion of XIAP in HCT116 colorectal carcinoma cells leads to diminished migration in scratch assays and XIAP-mediated cancer cell migration is RING domain-dependent. The same group also determined a role for XIAP–RhoGDI interaction via RING domain of XIAP to mediate cell motility by regulating actin cytoskeleton. XIAP was found to negatively regulate RhoGDI sumoylation at Lys-138 to promote cancer cell motility.^80, 81^

### Anti-migratory effects of IAPs in tumor cells

There have also been studies defining the anti-migratory effects of IAPs in tumor scenarios. We have shown that cIAP1, cIAP2, XIAP and ML-IAP can promote proteasomal degradation of C-RAF, a central kinase of the classical MAPK pathway that plays an important role in tumorigenesis downstream of Ras.^82, 83^ Knockdown of IAPs using siRNAs leads to enhanced lamellipodia and filopodia formation in HeLa cells as well as C-RAF-dependent enhanced cell migration. Oncogenic Ras mutations occur in ∼30% of all human cancers and C-RAF is pivotal to Ras signaling. C-RAF/MAPK pathway activation can promote cell survival by regulating expression/activity of BCL-2 family members.^84^ C-RAF can also directly restrain apoptosis by binding and inhibiting stress-induced kinase ASK-1.^85^ Furthermore, C-RAF can promote EMT, tissue invasion and metastasis by production of TGF*β* and inhibition of Rho effector, ROCK2.^86, 87, 88^

**Figure 3:**
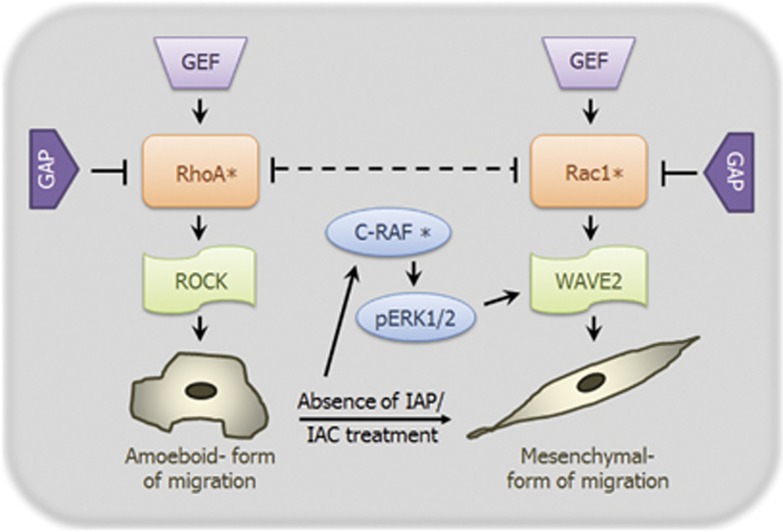
IAPs modulate cellular morphology and migration. Transient depletion of IAPs using siRNAs and IACs as well as cIAP1 and XIAP knockout MEFs show an elongated mesenchymal-like morphology and mesenchymal mode of migration (*indicates activation)

Further work in these lines have demonstrated that knockdown of IAPs using an IAC lead to an elongated morphology in various primary and tumor cells as well as Rac1- and MAPK-dependent enhanced migration in tumor cells. IAC-mediated knockdown of IAPs can lead to Rac1 stabilization and activation, leading to Rac1-dependent, enhanced mesenchymal-type of tumor migration^73, 89^ (Figure 3). This migration phenotype could be recapitulated by employing early passage cIAP1^−/−^ as well as XIAP^−/−^ MEFs. Knockout MEFs showed elongated morphology and migrated more efficiently as compared with strain-matched WT MEFs in a Rac1-dependent manner (Figure 3). Most of the discrepancy observed with respect to pro- and anti-migratory effects of IAPs could be attributed to the difference in the cell types employed, as many of the migration-regulating molecules controlled by IAPs could promote or inhibit migration in a context-dependent manner.

### Role of IAP-effector signaling in cell migration

The phenotype observed in our studies upon IAP knockdown using siRNAs and IACs resembles epithelial–mesenchymal transition (EMT) in the cells.^73^ EMT is an important cellular program occurring both during embryonic development as well as pathogenesis. Primary epithelial tumors can give rise to dissemination of single carcinoma cells by undergoing EMT, and thus, lead to tumor migration and metastasis.^90^ Pertinent to this, it was previously demonstrated that coordinated activation of Rac1 and the RAF/MAPK pathway is required for cell scattering and migration, key components of EMT upon activation of Ras in NBT II rat bladder carcinoma cell line.^91^ This is in line with our observations that coordinated activation of both C-RAF and Rac1 pathways leads to cell migration upon IAP depletion.

**Figure 4:**
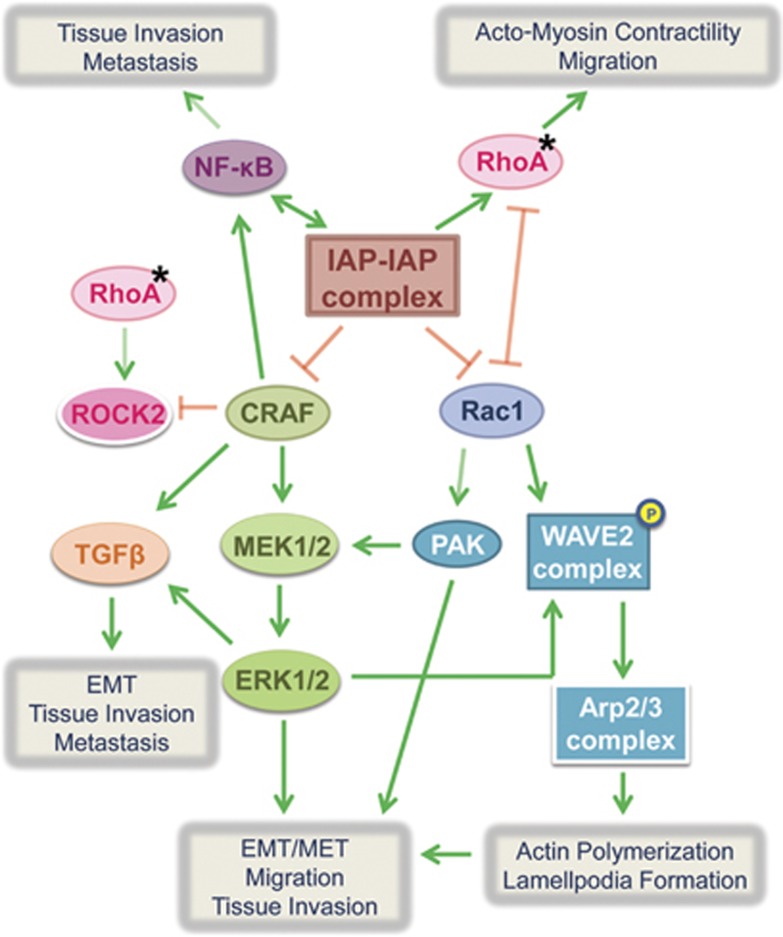
IAP-mediated signaling pathways involved in EMT/MET, cell migration, invasion and metastases. Shown is the representation of various cellular signaling pathways that are modulated by IAPs. IAPs regulate signal transduction via MAPK, RhoGTPase, NF-*κ*B and several other pathways to modulate the cellular migration at endogenous levels as well as under tumor progression conditions. The cross talk between these various pathways might influence the migratory ability of the cells in a context-/cell–type-dependent manner (*denotes activation)

Apart from these reports, there have been various instances demonstrating that these pathways potentiate the effect of each other. A recent report describes that ERK2, a downstream kinase of Ras-Raf-MEK-ERK pathway, can directly phosphorylate components of WAVE2 complex, WAVE2 and Abi1, essential for interaction of WAVE complex with Arp2/3 and actin to mediate actin polymerization and lamellipodia formation.^92^ Rac1 is the direct activator of WAVE complex and it is now shown that MAPK activation can potentiate its effect. Conversely, Rac1 has been shown to potentiate Ras-dependent skin tumor formation by hyperactivation of MEK-ERK1/2 signaling by Rac1 effector, Pak1.^93^ Furthermore, RacGEF Tiam1 has been implicated tumor invasiveness and metastasis.^94, 95, 96, 97^ Rac1 activation can promote both EMT and mesenchymal–epithelial transition (MET) by regulating assembly and disassembly of adherens junctions, and thereby has the ability to either prevent or facilitate tumor metastasis and secondary tumor formation.^98, 99, 100^ Rac1 activity is also vital for microtubule-induced FA disassembly for driving cell migration.^101^ Further, as previously stated, tumor cell plasticity is a major contributor towards metastasis. Rac1 activation drives mesenchymal mode of migration, which limits lung colonization in mice.^102^ Further, knockdown of IAPs can also lead to inactivation of RhoA.^73^ In addition, unpublished observations from our lab reveal a direct interaction between IAPs and RhoA independent of activation status of RhoA. Therefore, it necessitates a careful examination of coordinated Rac1-RhoA signaling pathways to predict the final migration phenotype in IAP-depleted cells (Figure 4).

Apart from C-RAF, Rac1 and RhoA, IAPs can also mediate both canonical and non-canonical NF-*κ*B pathways by ubiquitinating RIP and NIK kinases, respectively, at the interface of cell death/survival, inflammation and innate immune response as recently reviewed.^23, 24^ NF-*κ*B pathway can be activated due to TNF*α* secreted by activated macrophages, fibroblasts, Kupffer cells and a variety of tumor cells including breast carcinoma, colon carcinoma and B-cell lymphoma. Induction of NF-*κ*B and Akt signaling pathways by TNF*α* secretion inhibits GSK-3*β*-mediated phosphorylation of Snail and *β*-catenin, inducing their stabilization and nuclear localization.^103, 104^ Snail can interact with *β*-catenin and establish a positive feedback loop for Wnt-dependent transcription.^105^ Taken together, all the above signaling events contribute to angiogenic activity, accelerated EMT and tumor cell invasion and metastasis. NF-*κ*B activation can also directly promote tumor survival and metastases.^106, 107, 108, 109^ A recent study demonstrated a role for IAP-mediated regulation of non-canonical NF-*κ*B signaling in modulating migration and invasion in glioblastoma cells in a preclinical tumor model.^110^ As IAPs can directly regulate the NF-*κ*B pathway, they can either promote or inhibit tumor invasion and metastasis in a context-dependent manner. There has also been a report by Baumann *et al.*^111^ stating that C-RAF can synergize with another kinase, MEKK1 to activate NF-*κ*B pathway, which adds to the intricacy of these signaling complexes (Figure 4).

## Conclusion and Future Perspectives

Cell migration studies suggest a context-dependent role of IAPs in tumor cell migration. The final test for lethality associated with tumor is its metastatic potential or ability to migrate from the site of primary tumor. Nearly 90% of tumor lethality can be attributed to metastases. Therefore, it is vital to determine the direct role of IAPs in tumor metastases. Hwang *et al.*^112^ examined the role of XIAP in prostate cancer development using TRAMP (transgenic adenocarcinoma of mouse prostate) model. They checked for the tumor growth and metastatic potential in wild-type and XIAP-deficient, immunocompetent TRAMP mice and found that XIAP deficiency led to an aggressive form of disease without any compensatory increase in the levels of cIAP1 or cIAP2 levels.^112^ Therefore, it can be surmised that though there are elevated levels of XIAP in prostate cancers, it may not be responsible for carcinogenesis and metastasis. Along similar lines, another study was carried out by Mehrotra *et al.*^113^ to investigate the combined influence of XIAP and Survivin using a mouse metastasis model as well as *in vitro* matrigel invasion assay. They injected tumor cells from two adenocarcinoma cell lines, HCT116 (colorectal) and MCF-7 (breast) directly into the spleen to check for liver metastasis in immunocompromised SCID mice. Using cell lines, either stably overexpressing Survivin or having a stable knockdown of XIAP or both, they could show a higher metastatic potential of injected tumor cells being correlated to higher expression levels of IAPs.^113^ Both the aforementioned studies are carried out in mouse models with dissimilar background (immunocompetent *versus* immunocompromised) using adenocarcinomas of different origin as a basis for their study and showed contrasting results on XIAP-mediated tumor metastasis. This calls for further elucidation of pathways regulated by IAPs under diverse patho-physiological conditions.

Further, tumor microenvironment is a complex concoction of interacting factors and trying to mimic a human tumor *in vitro* or using *in vivo* mouse models including genetically engineered and humanized models might not be a true representation of the scenario. Though mouse models have contributed greatly to our current knowledge base and treatment strategies, recapitulating the stochastic nature of human tumorigenesis in a model organism is not completely possible.^114^ There are numerous examples of preclinically successful drugs failing to deliver clinically leading to loss of enormous amount of time, efforts and money. Therefore, it is vital that we exercise caution while extrapolating results from various tumor models.

IAPs are a part of complex signaling network, regulating components responsible for a diverse array of migration phenotypes. Deciphering the individual contribution of these components for a particular context (cell type/tumor type/stage) could determine the final fate of the cell. Therefore, it is also imperative that we recognize the underlying nature of the pathology for appropriate use of IAP-mediated cancer therapeutics. To improve the treatment efficacy of IAP antagonists, various combinatorial strategies for them including chemotherapy and radiotherapy have been tested pre-clinically.^24^ For instance, beneficial synergistic effects were observed when IAP antagonists are used in combination with death receptor agonists like TRAIL.^115^ Apart from this, recent study depicted combinatorial effect of Pak1, a downstream Rac effector, inhibition on IAP antagonist treatment in NSCLC cell lines, rendering these cells hypersensitive to apoptotic cell death.^116^ Development of combination therapy is being supported as it promotes better patient survival as shown recently in a metastatic breast cancer Phase III clinical trial.^117^ Combination therapy might promote synergistic effects leading to low drug dosage, as well as suppresses resistance to therapy if multiple cell survival pathways are targeted at once though there are chances of higher toxicity.^118^ Fortunately, clinical trials with IAP antagonists did not show any dose-limiting toxicity till date.^24^

In summary, a move towards IAP antagonist-based therapy should be pursued with caution as we still need better understanding of the complex signaling interplay in context of particular tumor types by using improved and multiple model systems. Further, as there might be a chance of enhanced cell migration and motility upon IAP inhibition in surviving tumor cells, enhancing the apoptotic potential and hampering the migratory potential of IAP antagonists by using cell death inducers and Pak1 antagonists, respectively, in combination promises to be a viable anticancer therapy.

## Figures and Tables

**Table 1:** IAPs and negative prognosis

**Cancer type**	**IAPs involved**	**Detection parameter**	**References**
*IAPs and negative prognosis*			
Esophageal squamous cell carcinoma	cIAP1 and cIAP2	Genomic DNA and RNA (11q21–23 amplicon)	Imoto *et al.*^119^
SCLC and NSCLC	cIAP1 and cIAP2	Genomic DNA and Protein (11q21–23 amplicon)	Dai *et al.*^27^
Hepatocellular carcinoma	cIAP1	Genomic DNA and RNA (11q21–23 amplicon)	Zender *et al.*^29^
MALT lymphoma	cIAP2	RNA (t(11 : 18)(q21 : 21) translocation)	Dierlamm *et al.*^120^
Acute myeloid leukemia (ALL)	cIAP2	RNA	Hess *et al.*^33^
Bladder cancer	XIAP	Protein and RNA	Li *et al.*^36^
	Nuclear cIAP1	Protein	Che *et al.*^38^
Colorectal cancer	XIAP	RNA and protein	Xiang *et al.*^37^
	cIAP2	Protein	Krajewska *et al.*^35^
Breast cancer	Nuclear XIAP	Protein	Zhang *et al.*^39^
Cervical sqamous cell carcinoma	Nuclear cIAP1	Protein (resistance to radiotherapy)	Imoto *et al.*^40^

Various IAPs reported to be upregulated in cancers and/or linked to negative tumor prognosis are listed

**Table 2:** IAPs and positive prognosis

**Cancer type**	**IAPs involved**	**Detection parameter**	**References**
*IAPs and positive prognosis*			
NSCLC	XIAP	Protein	Ferreira *et al.*^55^
Renal cell carcinoma	ML-IAP	Protein	Haferkamp *et al.*^57^
Pediatric ALL	ML-IAP	RNA and protein	Choi *et al.*^56^
Melanoma	ML-IAP	Protein	Lazar *et al.*^58^
Prostate cancer	XIAP	Protein	Seligson *et al.*^60^

Various IAPs reported to be downregulated in cancers and/or linked to positive tumor prognosis are listed

## Abbreviations

IAP: inhibitor of apoptosis
BIR: baculovirus IAP repeat
RING: really interesting new gene
UBA: ubiquitin-associated domain
UBC: ubiquitin conjugation domain
LRR: leucine-rich repeats
CARD: caspase recruitment domain
PCD: programmed cell death
NLR: NOD-like receptor
IBM: IAP-binding motif
IAC: IAP antagonist compound
MALT: mucosa-associated lymphoid tissue
ALL: acute myeloid leukemia
NSCLC: non-small cell lung carcinoma
RCC: renal cell carcinoma
TRAMP: transgenic adenocarcinoma of mouse prostate
GEF: guanine nucleotide exchange factor
GAP: GTPase-activating protein
RhoGDI: rho GDP-dissociation inhibitor
ECM: extracellular matrix
FA: focal adhesion
CGN: cerebellar granule neurons
EMT: epithelial–mesenchymal transition
MET: mesenchmal–epithelial transition
